# Unraveling the Potential of SGK1 in Osteoporosis: From Molecular Mechanisms to Therapeutic Targets

**DOI:** 10.3390/biom15050686

**Published:** 2025-05-08

**Authors:** Fei Yang, Changshun Chen, Rongjin Chen, Chenghui Yang, Zirui Liu, Lei Wen, Hefang Xiao, Bin Geng, Yayi Xia

**Affiliations:** 1 Department of Orthopaedics, Lanzhou University Second Hospital, Lanzhou 730030, China; 120220901491@lzu.edu.cn (F.Y.); chencs666@ynu.edu.cn (C.C.); 120220901411@lzu.edu.cn (R.C.); 120220901481@lzu.edu.cn (C.Y.); liuzt2023@lzu.edu.cn (Z.L.); wenl2023@lzu.edu.cn (L.W.); 120220901471@lzu.edu.cn (H.X.); ery_gengb@lzu.edu.cn (B.G.); 2 The Second Clinical Medical School, Lanzhou University, Lanzhou 730030, China; 3 Department of Orthopedics, Nanchong Central Hospital, Nanchong 637000, China; 4 Department of Orthopedics and Trauma Surgery, Affiliated Hospital of Yunnan University, Kunming 650032, China

**Keywords:** osteoporosis, SGK1, protein kinase, metabolic bone disease, therapeutic strategies

## Abstract

Osteoporosis (OP) is a prevalent metabolic bone disease, with several million cases of fractures resulting from osteoporosis worldwide each year. This phenomenon contributes to a substantial increase in direct medical expenditures and poses a considerable socioeconomic burden. Despite its prevalence, our understanding of the underlying mechanisms remains limited. Recent studies have demonstrated the involvement of serum glucocorticoid-regulated protein kinase 1 (SGK1) in multiple signaling pathways that regulate bone metabolism and its significant role in the development of osteoporosis. Therefore, it is of great significance to deeply explore the mechanism of SGK1 in osteoporosis and its therapeutic potential. In this paper, we present a comprehensive review of the structure and activation mechanism of SGK1, its biological function, the role of SGK1 in different types of osteoporosis, and the inhibitors of SGK1. The aim is to comprehensively assess the latest research progress with regards to SGK1’s role in osteoporosis, clarify its role in the regulation of bone metabolism and its potential as a therapeutic target, and lay the foundation for the development of novel therapeutic strategies and personalized treatment in the future. Furthermore, by thoroughly examining the interactions between SGK1 and other molecules or signaling pathways, potential biomarkers may be identified, thereby enhancing the efficacy of early screening and intervention for osteoporosis.

## 1. Introduction

Osteoporosis (OP) is a common metabolic bone disease whose key features include reduced bone mass and destruction of bone microarchitecture, which significantly increases the risk of bone fragility and fracture, particularly at high-prevalence sites such as the hip, spine, and wrist [1,2]. Currently, osteoporosis affects more than 200 million people worldwide, with approximately 8.5 million osteoporosis-related fractures occurring annually, resulting in a significant need for medical intervention and recovery [3]. Some studies have predicted that the number of people with osteoporosis will increase to approximately 300 million worldwide by 2025, leading not only to a dramatic increase in direct healthcare costs, but also to a further increase in indirect costs such as nursing, rehabilitation, and long-term care, which will cause a significant socioeconomic burden [4,5].

SGK1 is a member of the serine/threonine protein kinase family. Its initial identification was in rat breast cancer cells through a glucocorticoid-induced differential transcript expression screening [6]. It has been demonstrated that the expression of the SGK1 gene rapidly increases by a factor of 5–10-fold within a 30 min period under conditions where cells are stimulated by glucocorticoids or serum. This rapid increase in expression is therefore the reason why the gene is given the name serum- and glucocorticoid-regulated protein kinase 1 [7]. The full-length sequence of the gene is approximately 2.4 kilobases (kb) and encodes a protein kinase of 49 kilodaltons (kDa). SGK1 displays highly conserved properties from yeast to humans, and is widely expressed in various mammalian tissues and cell lines [8]. Recent studies have revealed that the regulatory mechanism of SGK1 is distinct from that of most protein kinases and can be activated by various stressors, including hypertonicity, hypotonicity, ultraviolet irradiation, heat shock, ischaemia, and trauma, as well as glucocorticoids, serum, and saline corticosteroid [9]. SGK1 may represent a functional intersection of multiple cellular signaling pathways and cellular phosphorylation cascade reactions [10]. Notably, SGK1 plays a pivotal role in the regulation of ion channels [11], cell proliferation [12], cell metabolism [13], cell survival [14], and apoptosis [15], as well as other signaling processes. Furthermore, SGK1 has been found to be closely related to a variety of physiological and pathological processes, including the regulation of renal function, bone metabolism, glucose regulation, and tumorigenesis, which have made it a research focus in the biomedical field [16].

Recent studies have demonstrated the pivotal role of SGK1 in the onset and progression of osteoporosis [13]. SGK1 exerts a substantial influence on multiple signaling pathways associated with bone metabolism, which significantly impacts osteoblast function and bone remodeling homeostasis [17,18]. In light of these findings, it is important to thoroughly investigate the mechanism of action and therapeutic potential of SGK1 in osteoporosis. Firstly, this review aims to comprehensively assess recent research progress on the role of SGK1 in osteoporosis pathology and elucidate its mechanism of action in osteoporosis, which will provide empirical support for exploring its key role in the regulation of bone metabolism and, in turn, provide basic data for clinical trials. Secondly, revealing the functional properties of SGK1 is expected to provide new targets for the development of novel therapeutic drugs against osteoporosis. In addition, by exploring the interactions of SGK1 with other molecules and signaling pathways, this review may also provide clues for the discovery of potential biomarkers, which may, in turn, facilitate the optimization of osteoporosis screening and early intervention strategies.

## 2. The Structure and Activation Mechanism of SGK1

**Figure 1:**
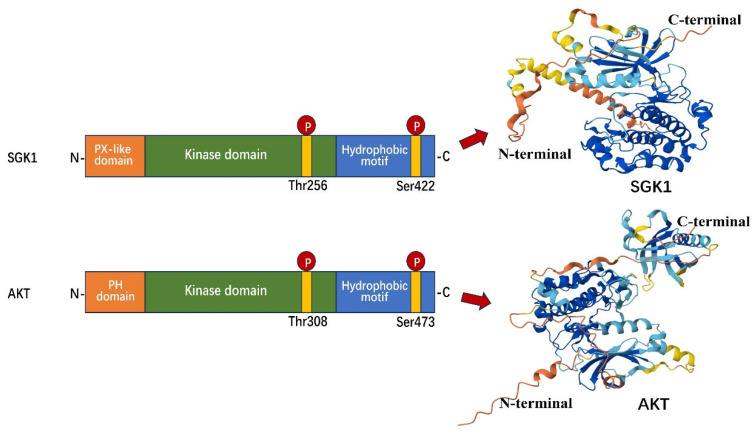
Domain organization (**left**) and 3D structure (**right**) of SGK1 and AKT. SGK1 and AKT share a similar domain organization consisting of an N-terminal domain, a catalytic domain, and a C-terminal domain. They also share similar phosphorylation sites: a Thr residue in the catalytic domain and a Ser residue in the C-terminal domain. SGK1: serum/glucocorticoid regulated kinase 1, AKT: protein kinase B.

SGK1 belongs to the SGK family, which consists of three members: SGK1, SGK2, and SGK3 [19]. The SGK isoforms are structurally very similar, with almost 80% sequence identity in the catalytic structural domain and about 50% sequence identity in the C-terminal regulatory region. The major structural difference between the three isoforms is at the N-terminus [20]. SGK is expressed in a number of species [21,22]. In mammals, SGK1 and SGK3 are widely expressed, but SGK2 expression is restricted to the liver, kidney, pancreas, and brain [10]. Although SGK1 lacks a PH structural domain, it has a high degree of structural similarity to protein kinase B (PKB/AKT) (Figure 1). In particular, its catalytic structural domain shares a homology of 54% with PKB/AKT, and both of them specifically recognize the RXRXX motif [8]. More interestingly, SGK1 has been found to possess two specific Ser/Thr regulatory sites: Thr256, located on the activation loop of its catalytic domain, and Ser422, situated at the C-terminal end. These sites exhibit a high degree of similarity to Thr308 and Ser473 of PKB/AKT [23]. A significant number of substrates of AKT have now been identified as substrates of SGK1 as well. These include glycogen synthase kinase-3 (GSK-3), the forkhead transcription factor member FKHR-1, the pro-apoptotic member of the Bcl-2 family BAD, Raf, IKK, CREB, and so on. However, it is important to note that the sites of phosphorylation of substrates by SGK1 and AKT are not identical. For example, both phosphorylate Thr32 of FKHRL-1, SGK1 phosphorylates Ser315 of FKHRL-1, and AKT phosphorylates Ser253 [9,20].

SGK1, an important effector molecule of the phosphatidylinositol 3-kinase (PI3K) signaling pathway, activates via a mechanism that is both significantly different from and similar to that of PKB/AKT. Both require phosphatidylinositol-dependent kinase (PDK)-mediated phosphorylation modification for full activation [22,24]. However, it is important to note that the activation of SGK1 is strictly dependent on the recognition of the PIF motif of PDK1. The Ser422 residue in the C-terminal hydrophobic domain binds specifically to the PIF motif of PDK1, which, in turn, facilitates the phosphorylation modification of the Thr256 site, resulting in the formation of a complete kinase-activated conformation. In contrast, the activation of PKB/AKT is less dependent on the PIF motif and exhibits a more flexible activation mode [24].

The divergent patterns of expression regulation further distinguish the activation properties of SGK1 from those of PKB/AKT, which is constitutively expressed in most cells, whereas SGK1 expression is significantly environmentally responsive, with transcript levels rapidly induced by cortisol hormones, gonadotropins, serum components, and a variety of stress signals [25,26]. These differences suggest that SGK1 may play a more dynamic role in cellular stress adaptation. In particular, the regulatory network of SGK1 breaks the paradigm of traditional kinases that rely only on post-translational modifications, and its expression is directly regulated by multiple signaling pathways at the transcriptional level, forming a dual transcriptional–post-translational regulatory loop [27]. Furthermore, the regulation of SGK1 activity is multidimensional: at the post-translational level, stimulation with epidermal growth factor (EGF) or insulin-like growth factor (IGF) triggers rapid phosphorylation and activation (with changes in kinase activity detected within minutes) [28]. At the level of protein stability, the ubiquitin ligase Nedd4-2 precisely controls intracellular SGK1 protein levels by mediating the ubiquitination modification of SGK1 at the K63 position, targeting and labeling it for entry into the 26S proteasome degradation pathway [29]. This balanced mechanism of rapid activation and timely degradation is important for maintaining the spatiotemporal specificity of SGK1 signal transduction.

With further research, molecules such as protein kinase C (PKC) [30], stress-activated protein kinase 2 (SAPK2/p38) [31], cyclic adenosine monophosphate (cAMP) [32], and the tumor suppressor protein p53 [33] have been successively found to be involved in the regulatory network of SGK1. These findings have not only extended the upstream regulatory spectrum of SGK1, but have also revealed its central role in physiopathological processes such as tumorigenesis (e.g., hepatocellular carcinoma) [34], embryonic development [35], maintenance of electrolyte homeostasis [36], and immunomodulation [37,38]. This provides an important experimental basis for the analysis of related disease mechanisms and the development of drug targets.

## 3. The Biological Function of SGK1

**Figure 2:**
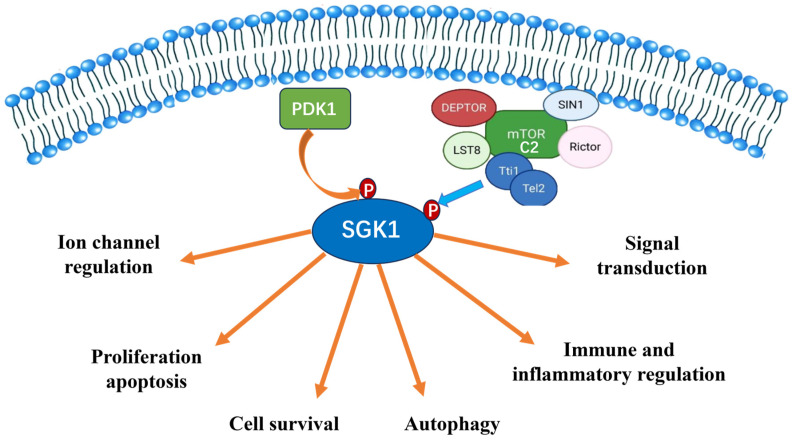
Biological functions of SGK1 related to osteoporosis. SGK1 plays an important role in a variety of physiological and pathological processes. However, we discuss in detail some critical functions of SGK1 related to osteoporosis here. PKD1: protein kinase D1, mTORC2: rapamycin complex 2.

As a pivotal integrator of cellular signal transduction networks and phosphorylation cascades, SGK1 exhibits distinctive unique kinase activity and a broad substrate profile, enabling it to fulfill a central regulatory function in diverse biological processes [10]. Despite the numerous biological functions attributed to SGK1, the present article will primarily focus on the biological functions of SGK1 in the regulation of ion channels, cell survival, immune and inflammatory regulation, signal transduction, and other related biological functions in the development of osteoporosis (Figure 2).

### 3.1. The Role of SGK1 in the Regulation of Ion Channels

SGK1, as a key regulator downstream of the aldosterone signaling pathway, plays an important role in the transport regulation of ion channels [36,39]. Its function is mainly reflected in the regulation of epithelial sodium channels (ENaCs) [40]. SGK1 prevents the ubiquitination and degradation of ENaCs by phosphorylating the ubiquitin ligase Nedd4-2 and inhibiting the binding of Nedd4-2 to ENaC [41]. This mechanism significantly increases the expression of ENaCs on the cell membrane surface and enhances the cellular uptake of sodium ions [42,43]. In addition, SGK1 is able to directly bind to the α and β subunits of ENaCs, further stabilizing the function and localization of ENaCs [44]. The role of SGK1 is not limited to sodium ion transport. It has been shown to regulate other ion channels, such as potassium and chloride channels, by similar mechanisms [33,45]. These aforementioned regulatory activities are important for maintaining sodium and potassium ion homeostasis in vivo and regulating cell volume and osmotic pressure [25,46]. Additionally, SGK1 has been demonstrated to augment the stability and surface activity of sodium-glucose cotransporter protein 1 (SGLT1), thereby markedly amplifying glucose uptake [47,48].

The aforementioned functions of SGK1 are closely associated with a variety of pathological processes, including hypertension, obesity, diabetes, and chronic kidney disease [49,50,51]. In such cases, ion channel dysfunction has been demonstrated to exacerbate the pathological processes, and modulation of SGK1 activity provides a novel idea for potential therapeutic targets in these diseases [52].

### 3.2. SGK1 Is Involved in Cell Proliferation, Apoptosis, and Cell Survival

SGK1 plays an important role in the regulation of cell cycle progression and its function is realized through dynamic localization between the nucleus and the cytoplasm [53]. Studies have shown that the subcellular distribution of SGK1 is regulated by the cell cycle and hormonal stimuli. During the G1 phase, SGK1 is mainly distributed in the cytosol in a hyperphosphorylated form, whereas it translocates to the nucleus during the S and G2/M phases [8], and this nucleoplasmic shuttling of SGK1 is essential for normal cell cycle progression [54]. Experiments that have manipulated the subcellular localization of SGK1 have indicated that maintaining constant levels of SGK1 in either the nucleus or the cytoplasm can inhibit cell growth and DNA synthesis. This finding serves to further illustrate the necessity of the dynamic localization of SGK1 in cell proliferation [12]. Furthermore, SGK1 has been demonstrated to regulate cell cycle progression in an indirect manner through the process of phosphorylating target proteins associated with both the cell cycle and survival [55,56]. In summary, SGK1 plays a critical role in cell proliferation and cell cycle processes through its dynamic nucleoplasmic localization, phosphorylation of forkhead box transcription factors, and regulation of other signaling pathways, which may be synergistically regulated with PKB [27,57].

The precise regulation of apoptosis, a highly conserved gene that controls the process of programmed cell death, is critical for maintaining tissue homeostasis and preventing pathological states. It has been demonstrated that SGK1 plays a pivotal role in the regulation of apoptosis through a dual regulatory network, functioning both as a direct promoter of cell survival signaling and as an indirect inhibitor of the initiation of the apoptotic pathway [58]. The kinase has been demonstrated to exhibit significant anti-apoptotic effects in a variety of cellular model systems, and its mechanism of action is closely linked to the regulation of transcription factor activity [59]. Molecular mechanistic studies have revealed that SGK1 performs its function by targeting the pro-apoptotic transcription of genes such as FOXO3a [60]. Specifically, SGK1 catalyzes specific phosphorylation modifications of FOXO3a, significantly impairing its DNA-binding capacity [61]. In conditions of insulin or growth factor deprivation, a decrease in SGK1 activity leads to an abnormal accumulation of FOXO3a in the nucleus, which, in turn, activates the transcription of pro-apoptotic genes, such as FasL and Bim, and triggers cell cycle arrest and activation of the mitochondrial apoptotic pathway [62]. Conversely, when SGK1 is activated by upstream signals, it undergoes nuclear translocation and binds to FOXO3a, inducing the latter to dissociate from chromatin and translocate to the cytoplasm through phosphorylation modification, resulting in a state of transcriptional inactivation [63,64]. This dynamic change in subcellular localization effectively blocks FOXO3a-dependent apoptotic gene expression and constitutes an important defense mechanism for cell survival [65].

Furthermore, the apoptosis regulatory spectrum of SGK1 is not limited to the transcription factor network. Recent studies have revealed that this kinase affects cell volume homeostasis and apoptotic signaling by regulating the activity of membrane proteins such as volume-regulated anion channels (VRACs), which further expands its functional dimension in cell fate determination [66]. These findings reveal that SGK1 forms a multilayered apoptotic defense system by integrating transcriptional regulation and signal transduction under stress conditions. In particular, during nutritional stress or genotoxic stimuli, SGK1 remodels the apoptotic threshold through a rapid response mechanism, providing a critical safeguard for cell survival [25].

### 3.3. SGK1’s Role in Regulating Immunity and Inflammation

SGK1 plays an important role in inflammation and immune regulation. It achieves its multiple biological effects by regulating the function of immune cells and inflammatory signaling pathways [67]. SGK1 has a significant regulatory effect on T cell differentiation [68]. SGK1 activates the m TORC1 signaling pathway and induces the expression of retinoic acid-related orphan receptor gamma (RORγ), which enhances the value-addition and differentiation of pro-inflammatory Th17 cells, while suppressing the generation of Treg cells, thereby promoting the development and maintenance of inflammation [69]. In the context of innate immunity, SGK1 has been observed to promote the expression of inflammatory factors (e.g., TNF-α and IL-1) in macrophages by activating the nuclear factor kappa-B (NF-κB) signaling pathway, thereby enhancing the intensity and duration of the local inflammatory response [70]. In the neutrophil inflammatory response, granulocyte–macrophage colony-stimulating factor (GM-CSF) has been shown to significantly upregulate SGK1 expression, thereby prolonging neutrophil survival [71]. Furthermore, SGK1 has been demonstrated to influence neutrophil migration and reactive oxygen species (ROS) generation by modulating Na+-K+ ion channels [72]. These processes are imperative for the eradication of pathogens and the regulation of both acute and chronic inflammation [67].

In recent years, as studies on the relationship between SGK-1 and inflammation have been carried out in different models and cellular systems, relevant studies have found that SGK1 can also regulate chronic inflammation through a negative feedback mechanism [73]. Specifically, it curbs excessive inflammatory responses by augmenting the expression of glucocorticoid-induced anti-inflammatory factors (e.g., IL-10) to forestall tissue damage and chronic inflammation [74]. Aberrant SGK expression is closely linked to a variety of inflammatory and immune diseases, including pulmonary fibrosis, inflammatory bowel disease (IBD), glomerulonephritis, and diabetes [75,76]. Notably, while SGK1 demonstrates pro-inflammatory or anti-inflammatory effects in various tissues, the underlying pathophysiological mechanisms remain to be fully elucidated. This further underscores the notion that SGK1 functions as a pivotal regulator of inflammation and immune-related diseases, thereby serving as a potential therapeutic target [77].

### 3.4. The Role of SGK1 in Cell Signaling and Regulation of Gene Expression

In the PI3K signaling pathway, SGK1, a pivotal effector molecule, plays a crucial role in regulating various biological processes, including cell proliferation, metabolism, and differentiation, in conjunction with PKB/AKT [78]. This signaling axis integrates stimulatory signals from extracellular growth factors and cytokines through the PI3K-PDK1/2-PKB/AKT cascade, triggering multiple biological responses, including DNA synthesis, cell survival, glucose transport, vesicular transport, receptor aggregation, and cytoskeletal remodeling [63]. It is noteworthy that recent studies have demonstrated that SGK1 exhibits more significant functional advantages in PI3K pathway-mediated physiological regulation [79]. In comparison with PKB/AKT, SGK1 demonstrates distinctive activation properties in response to diverse cellular stresses, including oxidative stress, osmotic pressure alteration, and nutrient deprivation. Its protein expression level and kinase activity can undergo rapid and substantial stress upregulation, while the expression and activation of PKB remain largely unaltered [9,80]. This discrepancy suggests that SGK1 may function as a specific effector of the PI3K signaling pathway during environmental adaptation and play a central regulatory role in the cellular stress response mechanism.

In the mitogen-activated protein kinase (MAPK) signaling pathway, SGK1 has been shown to exhibit a complex multi-target regulatory ability, precisely regulating the two core pathways, Ras-Raf-MEK-ERK and p38 MAPK, through kinase–substrate interactions and signaling cascade reactions [81]. Molecular mechanistic studies have demonstrated that SGK1 significantly inhibits the kinase activity of Raf kinase Ser364 by catalyzing its phosphorylation modification at the Ser364 site. This phosphorylation event constitutes a negative feedback regulatory loop of the Ras-Raf-MEK-ERK signaling pathway, which is physiologically important for preventing over-activation of this pathway under growth factor stimulation [82]. Furthermore, the regulatory spectrum of SGK1 is not limited to the ERK pathway. It has been demonstrated that SGK1 is deeply involved in the regulatory network of cellular stress response and inflammatory response through direct interaction with the core components of the p38 MAPK pathway, as well as indirect regulation of the c-Jun amino-terminal kinase (JNK) signaling module [83]. This cross-pathway regulatory pattern renders SGK1 a pivotal integration node of the MAPK signaling network, capable of coordinating biological processes such as cell survival, metabolic reprogramming, and immune response under different stress conditions [84].

These findings reveal the complex function of SGK1 as a signaling hub: it can act both as a sophisticated regulator of specific signaling pathways, and as a mediator of multi-pathway dialogues, playing a multidimensional regulatory role in maintaining cellular homeostasis and regulating pathophysiological processes. This multi-target regulation provides a theoretical foundation for understanding the function of SGK1 in tumorigenesis, metabolic diseases, and inflammation-related pathological processes.

## 4. The Role of SGK1 in Different Types of Osteoporosis

### 4.1. SGK1 and Postmenopausal Osteoporosis

**Figure 3:**
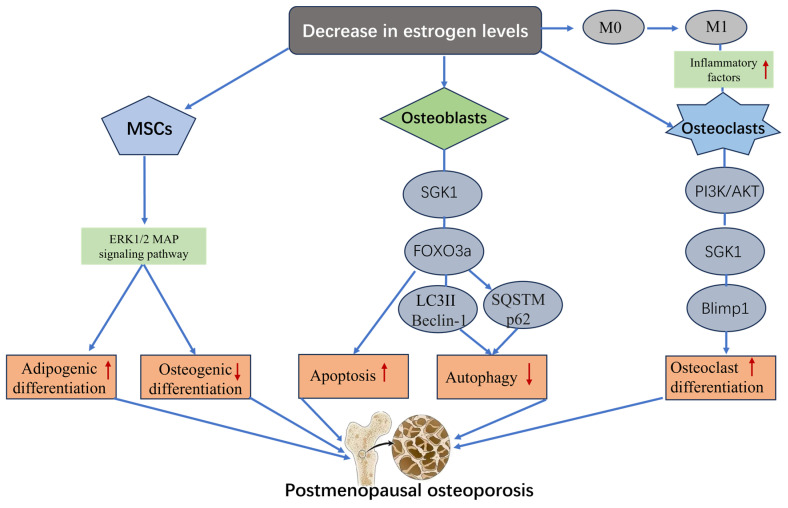
The signaling pathways describe the role of SGK1 in postmenopausal osteoporosis. The decrease in estrogen levels leads to SGK1 activation. Activated SGK1 leads to the occurrence and development of postmenopausal osteoporosis by inhibiting the osteogenic differentiation of mesenchymal stem cells and autophagy of osteoblasts, while promoting the apoptosis of osteoblasts and the formation of osteoclasts. MSCs: mesenchymal stem cells, M0/1: macrophages, FOXO3a: forkhead box O3a, LC3II: light chain 3, SQSTM/p62: sequestosome/protein 62.

Postmenopausal osteoporosis (PMOP) is a metabolic disease caused by estrogen deficiency that results in systemic bone loss and destruction of the bone microarchitecture, leading to increased bone fragility and increased fracture risk [85]. In recent years, with the revelation of the important role of SGK1 in estrogen signaling (Figure 3), the study of SGK1 in PMOP has gradually gained attention [17]. As mentioned above, SGK1 has a high degree of similarity to PKB/AKT and is widely involved in signaling pathways that regulate cell survival, proliferation, and metabolism. SGK1 has been shown to promote osteoblast survival and inhibit apoptosis through the PI3K/Akt pathway [86]. In PMOP, aberrant activation of the PI3K/Akt pathway is closely associated with decreased bone density [87]. In addition, SGK1 may also be involved in the regulation of osteogenic and osteoblastic homeostasis by modulating the ERK1/2 and p38MAPK pathways in the MAPK signaling pathway [88,89].

Patients with PMOP are often associated with a chronic low-grade inflammatory state, and inflammatory factors (e.g., TNF-α, IL-6) are thought to be important triggers of osteoclast activation [90]. Li et al. [91] found that TNF-α promotes osteoclast formation through activation of SGK1, which synergizes with RANKL, ultimately contributing to the osteoporosis syndrome in postmenopausal women. In addition, Lou et al. [92] showed, in OVX mice, that estrogen deficiency leads to macrophage polarization toward M1, a pro-inflammatory property, and that SGK1 indirectly affects the process of bone resorption by regulating the production and action of these inflammatory factors [18].

There remains considerable academic controversy surrounding the mechanism of SGK1 in PMOP. A number of studies have proposed that a decrease in estrogen levels can inhibit SGK1 kinase activity, thereby impairing osteoblast survival and accelerating apoptosis, which ultimately leads to bone loss [93]. However, other researchers believe that SGK1 is over-activated in PMOP patients, and the mechanism may involve two aspects: estrogen deficiency induces immune cells to differentiate towards a pro-inflammatory phenotype, and the secreted inflammatory cytokines promote osteoclastogenesis through activation of SGK1 [94,95]; and, at the same time, estrogen withdrawal directly inhibits bone metabolism pathways such as p38 MAPK, leading to an imbalance of bone resorption/formation [96]. Although existing studies have initially revealed the bidirectional regulatory potential of SGK1 in PMOP, the specific mechanism of SGK1 in the development of PMOP is still unclear, and most of the studies are limited to the cellular level and lack in vivo and clinical validation. It is therefore recommended that future studies focus on the interaction of SGK1 with estrogen receptors, the RANKL/OPG system, and other bone metabolism-related signaling pathways. Such studies should combine animal models and clinical cohort studies to elucidate the role of SGK1 in the pathological process of PMOP and to explore precise intervention strategies based on the regulation of SGK1.

### 4.2. SGK1 and Senile Osteoporosis

**Figure 4:**
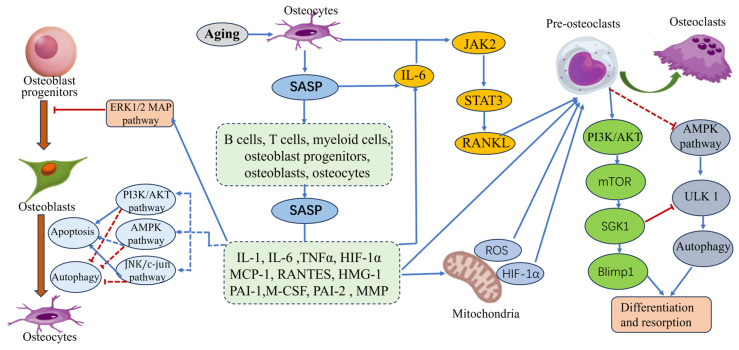
The signaling pathways describe the involvement of SGK1 in a series of important pathological processes in senile osteoporosis. In brief, osteocytes act on B cells, T cells, bone marrow cells, osteoblast progenitor cells, and osteoclasts in the bone microenvironment through the senescence-associated secretory phenotype (SASP), triggering a series of cascade chain reactions and secreting a large number of inflammatory factors and chemokines. By activating SGK1-related signaling pathways, these harmful cytokines further inhibit the proliferation and differentiation of mesenchymal stem cells and osteoblasts, promote mitochondrial oxidative stress and osteoclast maturation, and eventually lead to the development and formation of senile osteoporosis.

Primary osteoporosis is frequently classified into postmenopausal osteoporosis (type I), senile osteoporosis (type II), and idiopathic osteoporosis (adolescent). Senile osteoporosis (SOP) is widely recognized as osteoporosis that manifests in older adults over the age of 70 years [97]. With the accelerating process of global aging, SOP has emerged as a significant public health concern, exerting a substantial impact on the quality of life and health of older adults [98]. While the factors causing age-related bone loss are complex, recent findings indicate that disorders of bone metabolism due to abnormal activation of SGK1 (Figure 4) are closely related to the occurrence and development of SOP [25]. Therefore, a comprehensive assessment of the mechanism of the role of SGK1 in SOP may provide more of a theoretical basis and more practical possibilities for future research and treatment.

#### 4.2.1. Abnormal Activation of SGK1 by Inflammatory Factors

Aging is a vital life process for all living organisms. Bone senescence is chiefly marked by a reduction in bone mass, a decrease in cortical bone thickness, a decline in bone strength, and a decrease in the rate of bone mineralization [99]. The production of a senescence-associated secretory phenotype (SASP) by senescent cells contains numerous inflammatory cytokines. The abnormal activation of SGK1 by these inflammatory cytokines disrupts the balance between osteoblasts and osteoclasts, leading to increased bone resorption and decreased bone formation, and ultimately affecting the process of bone remodeling [100]. Furthermore, IL-6 has been found to increase osteoblast-mediated osteoclast differentiation through the activation of JAK2 and RANKL [101]. TNF-α has been observed to promote osteoclast formation by activating SGK1-mediated upregulation of Blimp1 expression through the PI3K/Akt pathway [102]. During the aging process, IL-17 secreted by T cells has been shown to inhibit bone formation by osteoblast progenitors and osteoclasts through aberrant activation of SGK1 [103]. Furthermore, a study by Farr et al. [100], measuring SASP markers in the bone marrow of young (6 months) and aged (24 months) rats, found that the expression of bone marrow cells, osteoblast progenitors, osteoclasts, and osteoblasts was significantly altered as the organism aged, and that the osteogenic capacity was diminished in aged rats with reduced bone formation. Consequently, the inhibition of the aberrant activation of SGK1 and the mitigation of the effects of inflammatory cytokines on bone metabolic homeostasis may emerge as a novel therapeutic approach for the treatment of senile osteoporosis in the future.

#### 4.2.2. Regulation of the Autophagy Process by SGK1

Basal levels of autophagy are critical for maintaining organismal homeostasis; however, abnormal levels of autophagy can result in various pathological conditions [104]. SOP is closely associated with autophagy, and the role of autophagy in SOP encompasses the following aspects. Firstly, autophagy regulates osteoblast (OST) metabolic homeostasis; autophagy promotes OST metabolic homeostasis by maintaining cellular metabolic balance and homeostasis, degrading and reutilizing harmful or obsolete cellular components, thereby reducing the risk of SOP [105]. Secondly, the process of autophagy (hereafter referred to as ‘autophagy’) has been demonstrated to play a pivotal role in the maturation and functional regulation of osteosarcoma (OS) cells. Autophagy has been shown to promote bone formation and repair by regulating the proliferation and maturation of osteosarcoma cells (OSCs) [106]. Thirdly, autophagy has been shown to play a crucial role in the regulation of osteoporosis-related factors. Autophagy has been demonstrated to regulate bone quality and homeostasis by regulating growth factors, inflammatory factors, and cell signaling pathways [107]. It is widely acknowledged that the mTOR signaling pathway serves as the primary regulator of cellular autophagy, with SGK1, a pivotal molecule within the mTOR signaling pathway, potentially playing a crucial role in the development and progression of SOP. Chen et al. [108] observed a decrease in autophagy levels in osteoblasts (OBs) and OSTs of aged mice. In a separate study, Onal et al. [109] demonstrated that autophagy plays a pivotal role in the regulation of SOP in mice. They observed that mice with specific knockout of autophagy-related genes and reduced expression of autophagy-related genes exhibited decreased bone mass and aging-related phenotypes in bone tissue. Furthermore, Wang et al. [110] demonstrated that nanohydroxyapatite could regulate the osteogenic differentiation of OBs by activating autophagy through the mTOR/SGK1 signaling pathway, and that this effect occurred in a dose-dependent manner. However, other studies have suggested that activation of autophagy promotes the function of osteoclasts (OCs), accelerates bone loss, and exacerbates osteoporosis. Fu et al. [111] showed that the autophagy level of OCs had an increased level of expression in individuals with glucocorticoid-induced bone loss. Ma et al. [112] conducted a study on RANKL-induced differentiation of OCs, in which they found that the administration of exogenous hydrogen sulfide (H2S) could inhibit the expression of autophagy levels through the mTOR signaling pathway, thereby inhibiting the differentiation function of OCs.

In summary, SGK1 has been demonstrated to have complex bidirectional functional properties in the regulation of skeletal homeostasis in patients with senile osteoporosis. In studies of inflammatory factors, aberrant activation of SGK1 has been found to significantly accelerate the progression of osteoporosis through pro-inflammatory factor-mediated enhancement of bone resorption. Interestingly, in autophagy-related studies, the regulatory effects of SGK1 on osteoblasts are dual: at basal levels of autophagy, it maintains osteoblast homeostasis by activating moderate autophagy; on the other hand, when pathological factors (e.g., oxidative damage, nutrient deprivation) induce autophagy over-activation, SGK1 accelerates osteoblast senescence by upregulating autophagy-associated proteins, resulting in a decrease in bone-forming capacity. This seemingly paradoxical phenomenon may be attributed to the following mechanisms: (1) Remodeling of the tissue senescence microenvironment: in elderly patients with osteoporosis, there is long-term, sustained release of inflammatory factors associated with senescent tissues and organs. These inflammatory factors lead to over-activation of autophagy, which results in an imbalance in the osteoclastic/osteogenic coupling, ultimately leading to the development of osteoporosis. (2) Mitochondrial dysfunction: autophagy protects osteoblasts by degrading damaged organelles in the physiological state, whereas excessive autophagy activation triggers mitochondrial dysfunction and apoptosis signaling, forming an ‘autophagy–senescence’ positive feedback loop. (3) Cross-regulation of signaling pathways: SGK1 may integrate bone metabolism pathways, such as PI3K/AKT, mTOR, and AMPK, to form a dynamic regulatory network in different pathological phases. Consequently, in subsequent studies, the construction of bone tissue- or cell-specific SGK1 knockout models, in conjunction with phosphorylated proteomics analysis, is recommended. This approach will facilitate further exploration of the mechanism of action between SGK1 and different signaling pathways. Conversely, the dynamic changes in the SGK1-autophagy axis in living animal models can be tracked by bioluminescence imaging, and dual regulatory strategies can be developed for the SGK1-autophagy axis to provide a staged and individualized intervention plan for age-related osteoporosis.

### 4.3. SGK1 and Diabetic Osteoporosis

Type II diabetes mellitus (T2D) is a pathological state characterized by insulin resistance, hyperglycemia, and subsequently impaired glucose-mediated insulin secretion [113]. Persistent hyperglycemia leads to multi-organ dysfunction, including in the skeletal system, where it is mainly characterized by disturbances in bone metabolism, bone loss, and progressive development of diabetic osteoporosis (DOP) [114]. This is one of the most serious complications in diabetic patients. Recent evidence emphasizes the important role of SGK-1 in diabetes and its complications [115]. In diabetes, elevated extracellular glucose concentrations induce glucocorticoid transcription [116]. When SGK1 in pancreatic B cells is stimulated by glucocorticoids, it reduces Ca^2+^ entry into B cells by upregulating voltage-gated K^+^ channels and inhibiting Ca^2+^ channel activity, which ultimately leads to reduced release of insulin [117]. An original study by Liu et al. [118] demonstrated that in the liver, SGK1, through the extracellular signaling-regulated kinase 1/2 (ERK1/2), regulated insulin sensitivity in mice. Inhibition of SGK-1 significantly ameliorated insulin resistance in glucosamine-treated HepG 2 cells and in the liver of db/db mice. These findings are consistent with a stress protein kinase response, as glucagon activation increases circulating glucose and decreases insulin release [119].

Obesity is a known risk factor for diabetes, and SGK1 has been identified as a contributing factor to the development of obesity [120]. SGK-1, a potent stimulator of the Na^+^-coupled glucose transporter SGLT1, has been shown to aberrantly activate SGLT1, thereby promoting intestinal glucose uptake and contributing to elevated blood glucose levels and adipose deposition [19]. Furthermore, SGK-1 has been shown to stimulate adipocyte differentiation and fat formation [121]. In addition, reduced glucose transport has been observed in adipocytes isolated from SGK-1^−^^/−^ mice. Further studies have demonstrated that SGK-1 can enhance glucose transporter-1 (GLUT1) activity by phosphorylating specific sites on GLUT1, as well as increase the abundance of transporter proteins in the plasma membrane, a critical process for glucose uptake by adipocytes [122]. Collectively, these findings indicate that SGK-1 plays a pivotal role in the development of obesity and T2D, which, in turn, contribute to increased morbidity and mortality in OP.

**Figure 5:**
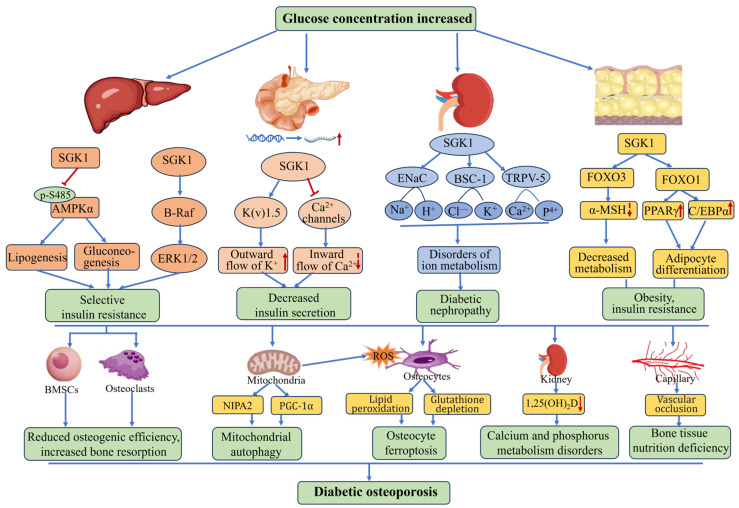
Related roles of SGK1 in diabetic osteoporosis. The high concentration of blood glucose in diabetic patients activates SGK1 in different organs, leading to decreased insulin secretion from the pancreas, insulin resistance in the liver, disorders of bone mineral metabolism in the kidney, abnormal accumulation of adipose tissue, and obesity. This series of pathological processes further promotes the progression of diabetes and osteoporosis. AMPKα: AMP-activated protein kinase, ENaC: epithelial sodium channel, BSC-1: Na^+^-K^+^-2Cl^−^ cotransporter, TRPV5: transient receptor potential vanilloid type 5, α-MSH: alpha-melanocyte stimulating hormone, PPARγ: peroxisome proliferator-activated receptor gamma, C/EBPα: CCAAT enhancer-binding protein alpha, NIP2: Non-Imprinted In Prader–Willi/Angelman Syndrome Region Protein 2, PGC-1α: peroxisome proliferator-activated receptor gamma coactivator 1-alpha.

Furthermore, SGK-1 has been associated with a multitude of complications in diabetic patients, including fluid retention, elevated blood pressure, abnormal coagulation, and vascular wall degeneration [52]. Targeting SGK-1 has been shown to reduce the incidence and delay the progression of diabetes, as well as to mitigate the severity of complications [123]. Consequently, SGK-1 may serve as a viable target for the development of novel therapeutic regimens for diabetes and diabetic osteoporosis (Figure 5).

### 4.4. SGK1 and Chronic Kidney Disease—Mineral and Bone Disorder (CKD-MBD)

**Figure 6:**
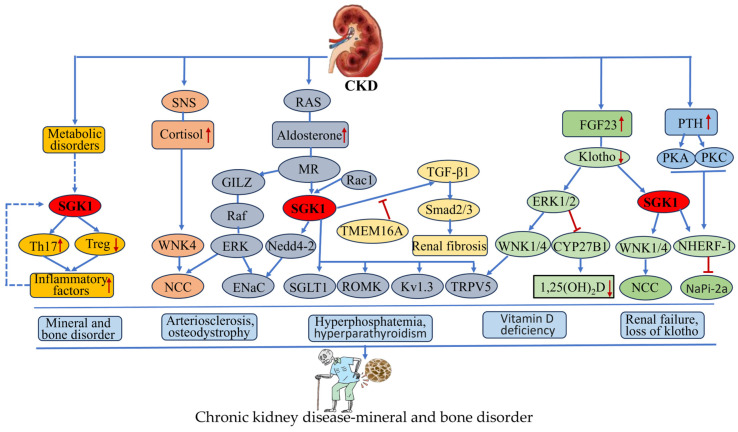
Pathogenic mechanisms of abnormally activated SGK1 in chronic kidney disease—mineral and bone disorder. Abnormal increases in aldosterone, cortisol, and parathyroid hormone and metabolic disorders in patients with chronic kidney disease activate SGK1 through multiple signaling pathways. The abnormal activation of SGK1 directly or indirectly regulates ion channels, T cell function, and 1α-hydroxylase, which leads to a series of pathological reactions, such as mineral bone disorder, osteodystrophy, vitamin D deficiency, and hyperparathyroidism, and finally leads to chronic kidney disease—mineral and bone disorder. SNS: sympathetic nervous system, RAS: renin–angiotensin–aldosterone system, GILZ: glucocorticoid-inducible leucine zipper, FGF23: fibroblast growth factor-23, PTH: parathyroid hormone, TMEM16A: transmembrane member 16A, ROMK: renal outer medullary potassium channel, NHERF-1: Na+/H+ exchanger regulatory factor-1.

Chronic kidney disease (CKD) is defined as a long-term, chronic disease that causes kidney damage due to various factors. The global prevalence of CKD is estimated to be 10–15%, making it one of the most prevalent non-communicable diseases [124]. Disorders of mineral metabolism, abnormal bone metabolism, and secondary osteoporosis have become the most common complications in patients with CKD, especially in patients with intermediate-to-advanced CKD [125]. Previous studies have confirmed that SGK-1 plays an important role in the progression of CKD-MBD [126]. The specific regulatory mechanisms are described in Figure 6. In CKD patients, there is increased secretion of aldosterone, due to elevated aldosterone levels resulting from the activation of the RAAS system. Aldosterone/MR stimulates SGK1 expression, which, further, causes renal injury and mineral bone disorder through inflammatory responses [127]. SGK1 also stimulates the secretion of TGF-β and accelerates renal fibrosis [128]. Zhou et al. found that the expression of SGK1 and TGF-β was enhanced in CKD rats, and aldosterone induced renal angiogenesis through the SGK-1/TGF-β signaling pathway, induced renal angiogenesis, and aggravated renal injury and calcium and phosphorus metabolism disorders in CKD [129]. In a similar vein, Sierra et al. [130] identified that heightened SGK1 activity was instrumental in the manifestation of salt corticosteroid-dependent renal injury in an SGK1 transgenic mouse model. The augmentation of SGK1 activity expedited corticosterone/salt-induced renal injury, in addition to calcium and phosphorus metabolism abnormalities and osteoporosis. In a related finding, Liu et al. [13] reported that decreased expression of p-ERK1/2 and p-SGK1 was accompanied by osteoporosis-like pathologic lesions in the femur in CKD-MBD model rats and NRK-52E cells cultured with TGF-β exposure. Fucoidan activated the ERK1/2-SGK1-NHERF-1-NaPi-2a pathway, thereby ameliorating kidney injury-associated calcium and phosphorus metabolism disorders and skeletal abnormalities in CKD-MBD model rats.

The most compelling physiological function of SGK-1 is the regulation of salt homeostasis. Under physiological conditions, SGK-1 phosphorylates Nedd4-2, thereby eliminating Nedd4-2-mediated tonic inhibition of epithelial sodium channels (ENaCs), which results in increased sodium reabsorption [131]. Additionally, SGK-1 can regulate ENaC activity through a mechanism that is independent of Nedd4-2 [132]. Conversely, Nedd4-2 phosphorylation has been shown to stimulate the degradation of SGK1 via the 26S proteasome [29]. This feedforward loop plays a pivotal role in the regulation of salt homeostasis in vivo. A series of related studies have further demonstrated that the regulatory effect of SGK1 on ion channels is not confined to ENaCs. SGK1 has also been found to regulate the activity of numerous ion channel transporter proteins, including TRPV5 [133], SCN5A [134], ROMK1 [135], and Kv1.3 [136]. These findings strongly suggest that SGK-1 plays a key role in the regulation of ion homeostasis in vivo. However, under pathological conditions, with the gradual progression of the degree of renal injury in CKD patients, SGK-1 in vivo is over-activated [137]. The over-activation of SGK1 has been shown to contribute to a variety of adverse outcomes, including renal fibrosis, lipid accumulation, and vascular dysfunction, which, in turn, can exacerbate metabolic dysregulation of calcium (Ca^2+^), phosphorus (P^4+^), parathyroid hormone (PTH), vitamin D, and fibroblast growth factor 23 (FGF23) [138]. This, in turn, can ultimately lead to the development of CKD-MBD in patients with CKD. Conversely, SGK-1 has been observed to promote an increase in Th17 cells and a decrease in Treg cells through the activation of the Foxo1/IL-23R pathway, resulting in an imbalance between the Th17/Treg cell subsets. Concurrently, the increased Th17 releases a substantial amount of pro-inflammatory factors, such as IL-6, IL-17, and TNF-α. These pro-inflammatory factors may be another significant factor contributing to abnormal bone metabolism and the development of osteoporosis [139].

Despite the relative abundance of information on the effects of SGK-1 on ion channels and CKD, most of the reported results presented only establish the relationship between SGK-1 and CKD. Fewer studies have been conducted on the relationship between SGK-1 and CKD-MBD, and the specific mechanisms are still not fully understood [13]. Consequently, further elucidation of the relationship between the two, as well as the development of relevant specific inhibitors, will be imperative for the prevention and treatment of chronic kidney disease osteoporosis in future studies.

## 5. The Potential of SGK1 as a Therapeutic Target for Osteoporosis

SGK1 plays a critical role in the pathophysiology of skeletal diseases, cancer, hypertension, diabetes, and cellular homeostasis [140]. As a potential regulator of transcription, enzyme activity, molecular activity, and signal transduction, SGK1 modulates the function of a variety of biological pathways through the activation of ion and protein channels, the maintenance of mitochondrial stability, and the regulation of immune responses [19]. SGK1’s involvement in numerous diseases and dysfunctions, both intra- and extracellular, underscores its critical role in biological processes. These functions include, but are not limited to, regulating cell survival, proliferation, differentiation, and function [141]. The development of inhibitors targeting SGK1 has emerged as a significant area of research in recent years, with notable results obtained [142]. Continued research in this field is anticipated to yield more inhibitors and a deeper understanding of SGK1’s regulatory functions. These inhibitors hold promise for applications in the treatment of various diseases, including cancer, hypertension, and osteoporosis [143]. The following section will focus on several SGK1 inhibitors that are currently under preclinical and clinical evaluation (Table 1).

### 5.1. GSK650394 and QGY-5-114-A

In 2008, Sherk et al. [144] reported the first instance of the SGK1 inhibitor GSK650394, a 7-azaindole derivative with two aryl substitutions at the C3 and C5 positions and 3-phenyl having carboxyl and cyclopentyl groups in the para- and meso-positions, respectively. The IC50 value of GSK650394 was determined to be 13 nM through the use of both the fluorescence polarization assay and the ATP mimetic assay [144]. In vitro activity assay results demonstrated that GSK650394 effectively inhibited the enzymatic activities of SGK1 and SGK2 with IC50 values of 62 nM and 103 nM, respectively. In a mitochondrial enzyme activity assay, GSK650394 exhibited reduced toxicity against M1 cells and HeLa cells [145]. Furthermore, the results of an in vitro kinase specificity assessment demonstrated that GSK650394 exhibited more than 30-fold higher selectivity for SGK1 compared to other members of the AGC protein kinase family; its selectivity is better than that of EMD638683 [146].

Increased bone resorption, caused by abnormal value-addition and differentiation of osteoclasts, is a primary factor in the development of osteoporosis, and the underlying cause may be related to the abnormal activation of SGK1 [147]. GSK650394 has been demonstrated to inhibit the abnormal activation of osteoclasts in several studies, without significant cytotoxicity. In vitro experiments have revealed that GSK650394 further inhibits the activation of NF-κB and MAPK signaling pathways through the inhibition of SGK1, thereby downregulating the expression of NFATc1 and RANKL. In vivo experiments have further confirmed that GSK 650394 treatment effectively improved bone mineral density in ovariectomy-induced bone loss mice [148]. In a recent study, Zhang et al. [18] utilized GSK 650394 to target SGK1, leading to a substantial retardation of osteoclastogenesis in vitro and a notable reduction in bone loss in mice with breast cancer bone metastases in vivo. Furthermore, Liang et al. [149] developed a new GSK650394 analog, QGY-5-114-A, which exhibited a lower IC50 value and significantly inhibited tumor cell growth, migration, and bone metastasis in rectal cancer. Unfortunately, the current study of QGY-5-114-A lacks in vivo data. Further in vivo evaluation of its toxicology, bioavailability, and other drug properties is needed. These findings collectively demonstrate that SGK1 may serve as a crucial molecular target for the future treatment of osteoporosis diseases.

### 5.2. EMD638683

EMD638683, a highly selective SGK1 inhibitor, was first identified in 2011 [150]. It has been demonstrated to inhibit SGK1 in a variety of pathologies, including hypertension, cancer, osteoporosis, chronic kidney disease, diabetes, and its associated secondary complications [141]. Currently, EMD638683 is undergoing clinical trials for the treatment of hypertension [49]. In multiple myeloma, complement C3a activates osteoclasts through the PI3K/PDK1/SGK1 pathway, and the inhibition of SGK1 using EMD638683 significantly reduces osteoclastogenesis, providing a new therapeutic target and strategy for the treatment of multiple myeloma patients [151]. In the context of diabetes, SGK1 upregulation has been observed to mediate mesenchymal transformation of vascular smooth muscle cells under hyperglycemic conditions, thereby enhancing medial vascular calcification. Furthermore, EMD638683 has been shown to significantly slow down the progression of hyperglycemia-induced vascular wall calcification [152]. However, related studies have shown that, in addition to SGK1, EMD 638683 also inhibits other targets, such as SGK2, SGK3, and mitogen- and stress-activated protein kinase 1 (MSK1) [153].

The drug EMD638683 has been shown to possess significant anti-inflammatory properties, playing a crucial role in the treatment of various inflammatory diseases [142]. In the context of angiotensin II-induced hypertension, the SGK1-FoxO1 signaling pathway has been identified as a pivotal mediator of the imbalance between Th17/Treg cell subsets and inflammatory responses in target organs. Conversely, treatment with the SGK1 inhibitor EMD638683 led to a significant reversal of the Th17/Treg imbalance and cardiorenal insufficiency [154]. In a recent study, Gan et al. [155] demonstrated that EMD638683 prevented angiotensin II-induced cardiac inflammation and fibrosis by blocking NLRP3 inflammatory vesicle activation through SGK1 inhibition. In addition, several studies have demonstrated that EMD638683 exerts anti-cancer effects and reduces tumor bone metastasis. In colon cancer cells (Caco-2), EMD638683 increased apoptosis by increasing caspase 3 activity and decreasing mitochondrial membrane potential, thereby promoting suicidal death of colon tumor cells [153]. A similar outcome was observed in rhabdomyosarcoma cells, where EMD638683 significantly inhibited tumor cell proliferation, migration, and survival, as well as reducing the drug resistance rate by inhibiting SGK1 expression [156]. In conclusion, EMD 638683 is one of the more desirable candidates for the treatment of SGK1-associated diseases, as no significant toxicity and off-target effects have been found. Nevertheless, EMD 63868 is poorly selected, and the majority of its reports remain at the cellular level. To enhance its therapeutic efficacy and reduce adverse effects, further improvements to the molecular structure of EMD 63868 are required, as well as in vivo studies to assess its therapeutic efficacy and adverse effects. The development of targeted drug delivery mechanisms and related studies will also contribute to the advancement of this field.

### 5.3. SI113 and 17a

SI113, a novel SGK1 inhibitor, was identified by D’Antona et al. [157] through high-throughput screening, and its biological effects were first reported, in 2015. Currently, SI113 has been studied mainly in various tumor models, with a focus on its antitumor effects, which are still in the preclinical research stage [158]. In the context of endometrial cancer, SI113 has been observed to markedly reduce the viability of endometrial cancer cells and to enhance apoptosis, while concomitantly activating endoplasmic reticulum stress by means of SGK1 inhibition [159]. In a separate study, Rango et al. [160] demonstrated the presence of potent anti-cancer activity in models of both hepatocellular carcinoma (HCC) and ovarian cancer through the modification of SI113’s motif. In the context of antitumor efficacy assessment experiments in HCC and ovarian cancer xenograft mouse models, a tumor volume reduction of more than 90% was observed; furthermore, no significant in vivo toxicity was observed. These studies have demonstrated that the inhibition of SGK1 by SI113 has good antitumor efficacy.

Recently, Halland et al. [142] designed another SGK1 inhibitor, 17a, based on the structure of 1H-pyrazolo[3,4-d] pyrimidine. To optimize the ADMET and selectivity profiles of the compound, several ligand-based computer models were applied. In animal experiments, it was demonstrated that this novel inhibitor satisfies the safety and pharmacokinetic requirements for oral administration, and it exhibits high SGK1 selectivity and adequate bioavailability. 17a is anticipated to be another novel drug for the treatment of osteoarthritic diseases.

The aforementioned studies have adequately demonstrated that SI113 and its derivatives possess favorable safety and biological activity profiles, thus establishing them as optimal SGK1 inhibitors. Unfortunately, there is a paucity of studies conducted on anti-osteoporosis models, which may be a primary focus area for future research endeavors.

### 5.4. ZINC00319000

ZINC00319000, a novel SGK1 inhibitor, was selected from the ZINC database through virtual high-throughput screening. Subsequently, Mohammad et al. [161] employed virtual screening, molecular docking, and interaction analyses to comprehensively evaluate its affinity with SGK1, specificity, and safety. The findings indicated that ZINC00319000 exhibited a stable binding affinity for the SGK1 structure, accompanied by a favorable safety and specificity profile, positioning it as a promising candidate for further development as an SGK1 inhibitor. Regrettably, there is a paucity of studies addressing ZINC00319000, and there is an absence of pertinent in vitro and in vivo biological assessments. Hence, there is a necessity for the future investigation of this compound, encompassing the characterization of its structure, its potential modification, if deemed necessary, and the subsequent comprehensive evaluation of its safety and efficacy.

### 5.5. Herbacetin (HBT)

Herbacetin, a flavonoid extracted from Ephedra and Sedum, has been the subject of numerous studies, which have reported its diverse biological functions, including its potential to improve insulin resistance, reduce inflammation, and exhibit antioxidant and antitumor properties [162,163]. Earlier studies primarily focused on the inhibitory properties of HBT on AKT, ACLY (adenosine triphosphate-citrate lyase), and FBPase (fructose 1,6-bisphosphatase) [164]. Due to the similarity between SGK1 and AKT, researchers have begun to explore its role as an inhibitor of SGK1. Through the use of mass spectrometry, kinase inhibition assay, thermal shift assay, and molecular docking, it has been demonstrated that HBT can directly bind to and inhibit SGK1 [165]. Further studies have shown that HBT significantly inhibits cardiomyocyte hypertrophy and reduces reactive oxygen species (ROS) synthesis in vitro and in vivo by inhibiting the phosphorylation of SGK1, thereby preventing the activation of its downstream molecule FoxO1 [166]. However, the bioavailability of HBT is low and has been less commonly reported as an SGK1 inhibitor. Future studies should focus on improving bioavailability, further investigating its pharmacological mechanism, and evaluating its safety and optimal dose.

## 6. Summary and Outlook

SGK1 exerts a regulatory influence on multiple biological pathways by activating various ion channels [167] and protein kinases [8], maintaining mitochondrial stability [168], and modulating immune responses [169]. In addition to these functions, SGK1 plays a pivotal role in regulating the signaling pathways of bone metabolism in various types of osteoporosis, thereby affecting the function of bone-associated cells and the balance of bone remodeling [13,17]. Consequently, SGK1 emerges as a promising molecular target for therapeutic intervention in osteoporosis diseases.

Synthesis of the data from previous studies reveals that, in cases of senile osteoporosis [25], diabetic osteoporosis [117], and chronic kidney disease—mineral and bone disorder [138], SGK1 demonstrates elevated expression levels and abnormal activation. However, in postmenopausal osteoporosis, divergent results have been reported by various researchers. Further exploration of the molecular mechanism of SGK1 in osteoporosis and its application in the treatment of specific types of osteoporosis, especially in postmenopausal osteoporosis, is needed in the future.

Despite the value of SGK1 as a potential therapeutic target having been revealed by existing studies, the development of SGK1-targeted therapies that combine utility, safety, and the absence of toxic side effects still faces multiple challenges [141]. Meanwhile, these inhibitors are currently used mainly in oncological diseases, with relatively few reports on their use in osteoporosis. In light of the current research progress, three major technical bottlenecks must be addressed: 1. The molecular design of specific inhibitors: The structural domain of SGK1 exhibits a high degree of homology with other members of the AGC kinase family, resulting in poor specificity of existing inhibitors and a consequent propensity for off-target effects. Furthermore, the challenge of balancing target binding affinity and metabolic stability remains a key challenge in medicinal chemistry. 2. Construction of tissue-specific delivery systems: Abnormal activation of SGK1 in the bones, kidneys, and central nervous system is closely related to the pathological processes of osteoporosis, diabetic nephropathy, and Alzheimer’s disease. Ideal inhibitors must possess precise modulation of tissue penetration, for example, through nanoparticle surface modification of bone-targeting peptides, or targeted activation in pathological microenvironments using prodrug strategies. The employment of tissue-targeted delivery systems has the potential to enhance drug concentration within target tissues, while concomitantly reducing the risk of peripheral tissue exposure. 3. Optimization of drug resistance and stabilizing efficacy: Previous studies have shown that long-term SGK1 inhibition tends to induce compensatory activation of the AKT pathway, leading to therapeutic resistance or attenuation of efficacy. In the future, further chemical modification of the inhibitor structure or the maintenance of steady-state blood levels by controlled release techniques (e.g., PLGA microspheres) will be required.

As a common pathological hub of multiple diseases, the development of SGK1 inhibitors necessitates the integration of the multidisciplinary strengths of biology, medicinal chemistry, and nanomedicine. Future research should focus on the establishment of better high-throughput screening platforms, the development of intelligent delivery systems with ‘on/off’ functions, and the creation of a system for dynamic monitoring of drug concentration in tissues. The implementation of these innovative strategies is expected to facilitate the translation of SGK1-targeted therapies from the laboratory to the clinic, and provide new solutions for the treatment of metabolic bone diseases, diabetes, hypertension, and tumors.

In summary, further exploration of the function of SGK1 in osteoporosis and its potential as a therapeutic target is warranted. These studies have the potential to yield novel therapeutic strategies and further elucidate the mechanisms underlying this prevalent bone metabolic disease.

## Figures and Tables

**Table 1:** Inhibitors of serum glucocorticoid-regulated kinase-1 and their chemical structures.

Inhibitor	Structure	IC_50_	Pathways Targeted	Disease	Ref.
GSK650394 (C_25_H_22_N_2_O_2_)	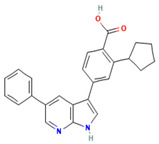	62 nM	mTOR-FoxO3 pathway PI3K-Akt signaling	Cancer Osteoporosis Age-related diseases Diabetes Inflammation	[144,145,146,147,148]
QGY-5-114-A	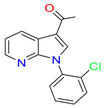	122.9 μM	ND	Colorectal cancer Metastatic bone tumor	[149]
EMD638683 (C_18_H_18_F_2_N_2_O_4_)	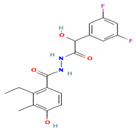	3 μM	NF-κB signaling pathway	Colorectal tumor Osteoporosis Hypertension Diabetes	[49,141,150,151,152,153,154,155,156]
SI113 (C_23_H_24_N_6_O)	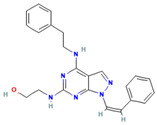	600 nM	Caspases-PARP signaling pathway	Ovarian cancer Hepatocellular carcinoma Endometrial cancer Osteoporosis	[157,158,159,160]
17a (C_23_H_21_ClFN_5_O_4_S)	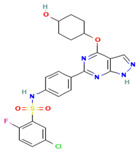	NA	ND	Hepatocellular carcinoma Glioblastoma	[142]
ZINC00319000 (C_19_H_15_O_6_)	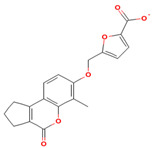	NA	ND	Cancer Metastatic bone tumor	[161]
Herbacetin (C_15_H_10_O_7_)	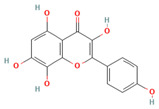	750 nM	mTOR-FoxO3 signaling pathway ROS production	Hepatocellular carcinoma Osteoporosis Cardiac hypertrophy Diabetes	[162,163,164,165,166]

## Data Availability

Not applicable.
